# Triaceto­nitrile­(1,4,7-trimethyl-1,4,7-tri­aza­cyclonona­ne)cobalt(II) bis­(tetra­phenyl­borate)

**DOI:** 10.1107/S241431462400539X

**Published:** 2024-06-11

**Authors:** Jeongcheol Shin, Jin Kim, Jonghoon Choi

**Affiliations:** a Department of Chemistry Duksung Women’s University Seoul 01369 Republic of Korea; b Department of Chemistry, Sunchon National University, Sunchon 57922, Republic of Korea; c Department of Chemistry Education, Chonnam National University, Gwangju 61186, Republic of Korea; University of Antofagasta, Chile

**Keywords:** crystal structure, cobalt, tacn, octa­hedral geometry

## Abstract

The title cobalt(II) complex, [Co(C_2_H_3_N)_3_(C_9_H_21_N_3_)](C_24_H_20_B)_2_ or [(tacn)Co(NCMe)_3_][BPh_4_]_2_, has been characterized by single-crystal X-ray diffraction. It incorporates the well-known macrocyclic tacn (1,4,7-trimethyl-1,4,7-tri­aza­cyclo­nona­ne) ligand, which is coordinated facially to the metal center. The complex crystallizes in space group *P*2_1_/*c* with *Z* = 4. The divalent cobalt ion exhibits a six-coordinate octa­hedral geometry by one tacn and three aceto­nitrile ligands. Two non-coordinating tetra­phenyl­borate (BPh_4_^−^) anions are also present.

## Abstract 2

The title complex exhibits a distorted octa­hedral geometry about the cobalt centre. The divalent cobalt ion is surrounded by three aceto­nitrile solvento ligands and one tridentate tacn ligand.

## Graphical abstract

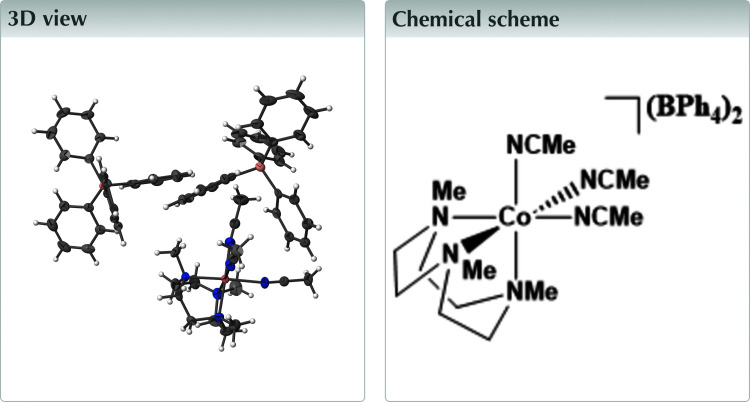

## Structure description

Cobalt complexes have attracted much attention due to their applications as catalysts for hydrogenation and hydrogen evolution reactions (Lin *et al.*, 2017 ▸; Zhang *et al.*, 2013 ▸, 2017 ▸). A rational design of catalyst is essential for the development of efficient cobalt catalysts. A scorpionate ligand allowing the facial coordination to a metal ion leads to the high-spin electronic configuration in low-coordinate cobalt complexes (Detrich *et al.*, 1996 ▸; Cordeiro *et al.*, 2021 ▸; Gu *et al.*, 2023 ▸). Particularly, such a high-spin state of a monovalent cobalt ion allows the oxidative addition of di­hydrogen, generating the cobalt dihydride, which is an important inter­mediate for the aforementioned catalyses. The 1,4,7-trimethyl-1,4,7-tri­aza­cyclo­nonane (tacn) ligand exhibits an almost identical coordination mode with scorpionate ligands and it is proposed that a metal complex supported by tacn can display similar chemical and catalytic properties. Although tacn has also been introduced to cobalt, most of the resulting complexes show binuclear geometry. This study shows that [(tacn)Co(NCMe)_3_][BPh_4_]_2_ is monomeric.

This report describes the preparation and the crystal structure of [(tacn)Co(NCMe)_3_][BPh_4_]_2_ (**1**), which is a potential pre-catalyst. Compound **1** was prepared by the sequential reaction of the solution of cobalt(II) bromide (CoBr_2_) in aceto­nitrile with 1 equiv. of tacn and 3 equiv. of sodium tetra­phenyl­borate (NaBPh_4_). As a result of the paramagnetic character of the cobalt cation, the ^1^H NMR spectrum exhibits paramagnetically shifted peaks at 177.0, 48.3, 48.3, and 1.93 p.p.m. and the diamagnetic tetra­phenyl­borate anions can be assigned at 7.18, 6.83, 6.81, 6.79, 6.69, 6.67, and 6.65 p.p.m. (see Figure S1). The presence of the non-coordinating BPh_4_^−^ anion was also confirmed by ^11^B resonance at −6.78 p.p.m. (see Figure S2).

**Figure 1:**
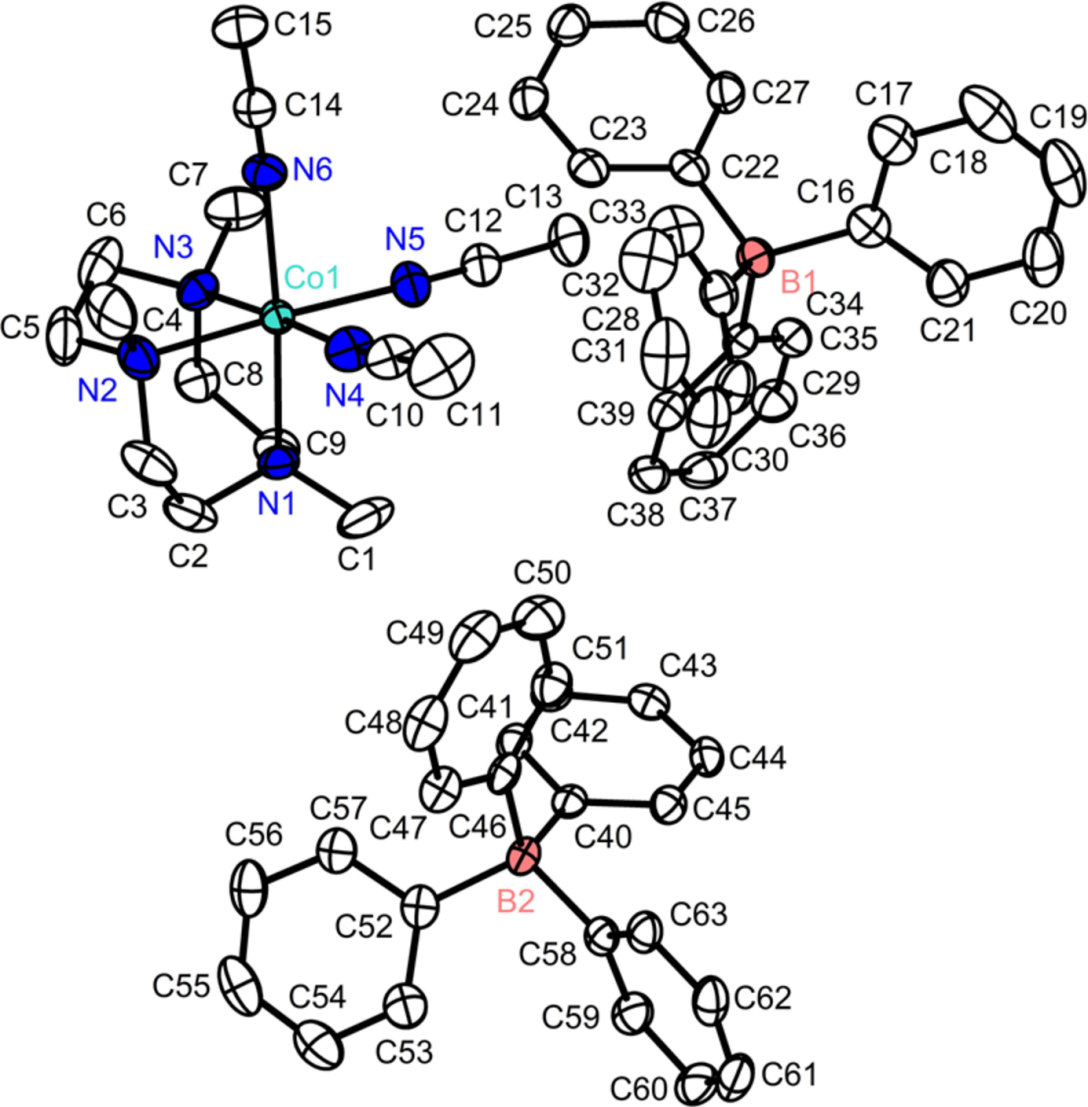
X-ray crystal structure of **1** (ellipsoids at 50% probability). All hydrogen atoms are omitted for clarity.

**Figure 2:**
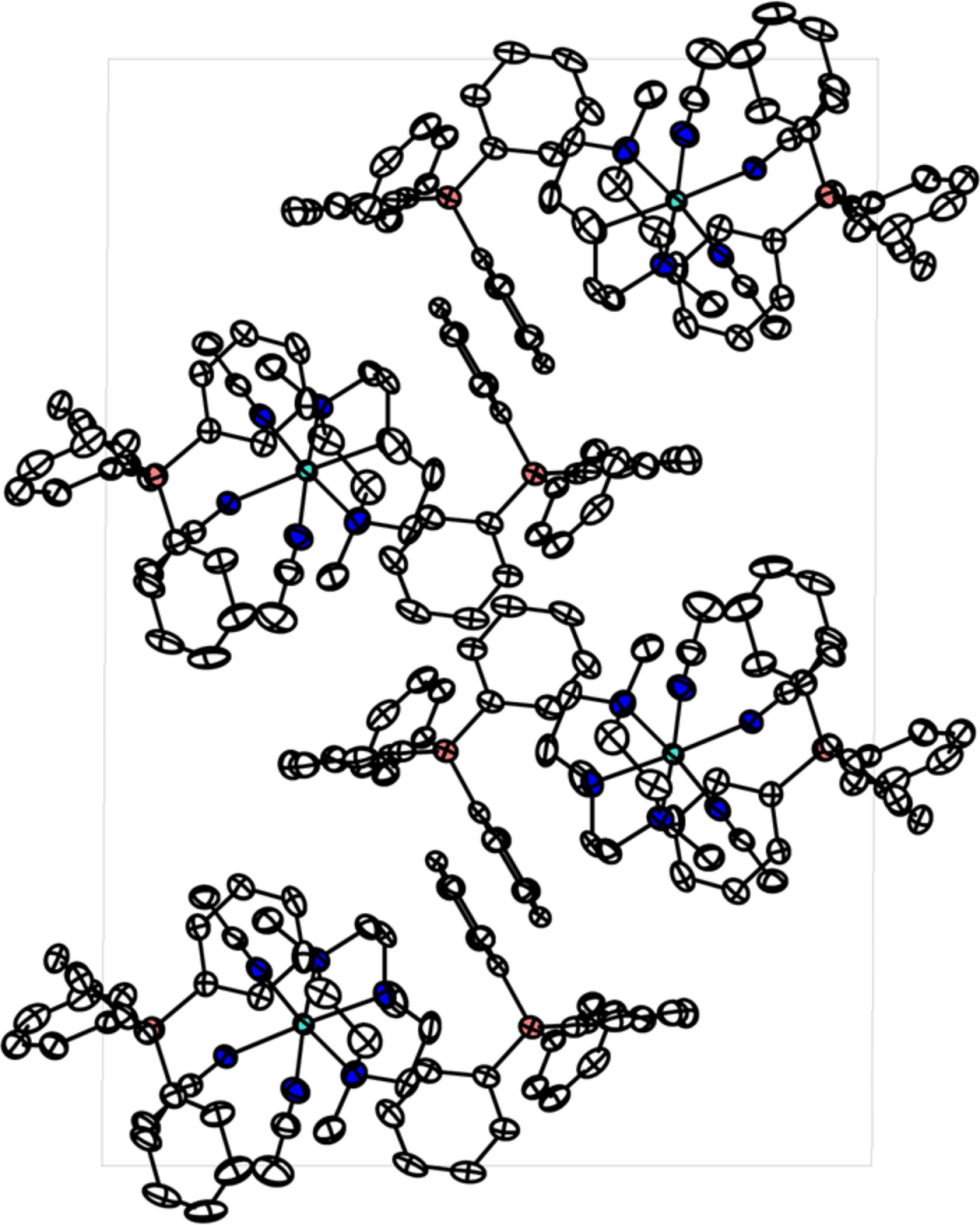
Crystal Structure of **1** in a view along the crystallographic *b*-axis direction. All hydrogen atoms are omitted for clarity.

The single-crystal X-ray diffraction data reveals that the divalent cobalt ion adopts an octa­hedral geometry with six nitro­gen donors of tacn and three aceto­nitrile ligands with two non-coordinating BPh_4_^−^ ions (see Fig. 1 ▸). The tacn ligand is coordinated to the cobalt(II) center in the facial coordination fashion, exhibiting N_tacn_—Co1—N_tacn_ bond angles of 83.16 (9), 82.86 (9) and 83.18 (9)°. The solvent ligands, aceto­nitrile, are also coordinated to cobalt in a *cis* manner. The three N_tacn_—Co1—N_aceto­nitrile_ bond angles are 175.23 (9), 175.58 (9) and 176.62 (10)°, clearly showing the octa­hedral geometry of **1** (Table 1 ▸). The Co—N bond lengths ranging from 2.094 (3) to 2.153 (2) Å indicate that the high-spin divalent cobalt ion is supported by six l-type nitro­gen donors (Kershaw Cook *et al.* 2013 ▸). This result corresponds to the ^1^H NMR spectrum showing paramagnetic character. In the crystal, the discrete cobalt complexes and BPh_4_^−^ anions are arranged along the *b*-axis direction (see Fig. 2 ▸). There are no directional inter­molecular inter­actions or hydrogen bonding among mol­ecular ions.

A search in the Cambridge Structural Database for structure **1** did not reveal any reported structures, including derivative searches. Similar dimeric cobalt compounds supported by tacn have been reported (Bossek *et al.* 1997 ▸; Thangavel *et al.* 2013 ▸) but a monomeric cobalt complex has not previously been structurally characterized.

## Synthesis and crystallization

**Experimental details**

Cobalt(II) bromide (CoBr_2_), tacn, and sodium tetra­phenyl­borate (NaBPh_4_) were purchased from Sigma Aldrich. All manipulations were carried out using standard glovebox techniques under N_2_ atmosphere. Unless otherwise noted, solvents (THF and aceto­nitrile) were de­oxy­genated and dried by 4 Å mol­ecular sieve. Tetra­hydro­furan (THF) was tested with a standard purple solution of sodium benzo­phenone ketyl in THF in order to confirm effective oxygen and moisture removal.

**Figure 3:**
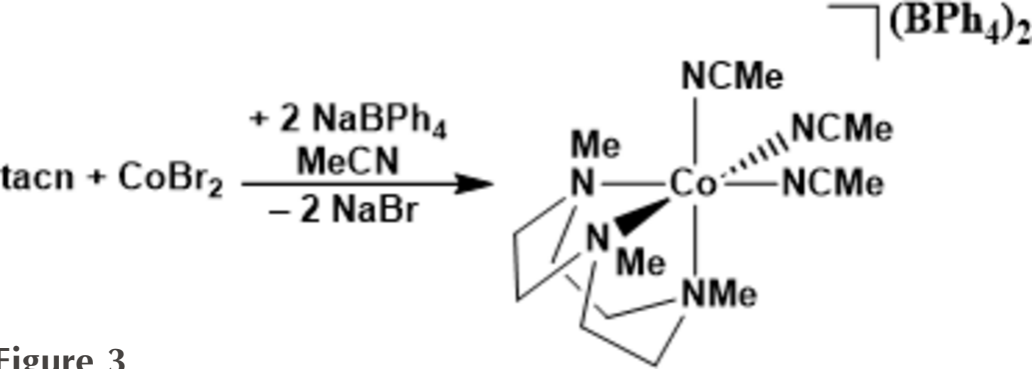
Reaction scheme.

**[(tacn)Co(NCMe)_3_][BPh_4_]_2_ (1).** The reaction scheme is shown in Fig. 3 ▸. To a solution of CoBr_2_ (318 mg, 1.44 mmol) in 5 ml of THF, a solution of tacn (254 mg, 144 mmol) in 5 ml of THF was added dropwise and the reaction mixture was stirred at room temperature for 1 h. The purple precipitate formed was dried under vacuum. The reaction mixture was dissolved in 10 ml of MeCN and NaBPh_4_ (1.486 g, 4.321 mmol) was added. The reaction mixture was stirred at room temperature for 3 d then filtered through Celite and the solution was dried under vacuum. The compound [(tacn)Co(NCMe)_3_[BPh_4_]_2_ (**1**, 1.258 g, 1.268 mmol, 88.0% yield) was isolated as a pale-orange solid after washing with a minimum amount of MeCN. X-ray quality crystals were grown by cooling down of a saturated solution of **1** in aceto­nitrile at −35° C. ^1^H NMR (DMSO-*d*_6_, 400 MHz): δ 177.0, 48.3, 48.3, 7.18, 6.83, 6.81, 6.79, 6.69, 6.67, 6.65, 1.93 p.p.m.. ^11^B NMR (DMSO-*d*_6_, 128 MHz): δ −6.78 p.p.m..

## Refinement

Crystal data, data collection and structure refinement details are summarized in Table 2 ▸.

## Supplementary Material

Crystal structure: contains datablock(s) I. DOI: 10.1107/S241431462400539X/bx4029sup1.cif

Structure factors: contains datablock(s) I. DOI: 10.1107/S241431462400539X/bx4029Isup2.hkl

supporting information. DOI: 10.1107/S241431462400539X/bx4029sup3.pdf

CCDC reference: 2310790

Additional supporting information:  crystallographic information; 3D view; checkCIF report

## Figures and Tables

**Table 1:** Selected geometric parameters (Å, °)

Co1—N1	2.143 (2)	Co1—N4	2.129 (3)
Co1—N2	2.139 (2)	Co1—N5	2.094 (3)
Co1—N3	2.141 (2)	Co1—N6	2.153 (2)
			
N1—Co1—N2	83.16 (9)	N1—Co1—N6	175.58 (9)
N1—Co1—N3	82.86 (9)	N2—Co1—N5	175.23 (9)
N2—Co1—N3	83.18 (9)	N3—Co1—N4	176.62 (10)

**Table 2:** Experimental details

Crystal data	
Chemical formula	[Co(C_2_H_3_N)_3_(C_9_H_21_N_3_)](C_24_H_20_B)_2_
*M*_r_	991.80
Crystal system, space group	Monoclinic, *P*2_1_/*c*
Temperature (K)	133
*a*, *b*, *c* (Å)	18.1245 (16), 11.6689 (10), 26.067 (2)
β (°)	90.332 (2)
*V* (Å^3^)	5513.0 (8)
*Z*	4
Radiation type	Mo *K*α
μ (mm^−1^)	0.36
Crystal size (mm)	0.10 × 0.09 × 0.05

Data collection	
Diffractometer	Bruker APEXII CCD detector
Absorption correction	Multi-scan (*SADABS*; Krause *et al.*, 2015 ▸)
*T*_min_, *T*_max_	0.627, 0.745
No. of measured, independent and observed [*I* > 2σ(*I*)] reflections	112545, 9437, 6984
*R*_int_	0.096
(sin θ/λ)_max_ (Å^−1^)	0.589

Refinement	
*R*[*F*^2^ > 2σ(*F*^2^)], *wR*(*F*^2^), *S*	0.056, 0.107, 1.14
No. of reflections	9437
No. of parameters	655
H-atom treatment	H-atom parameters constrained
Δρ_max_, Δρ_min_ (e Å^−3^)	0.59, −0.63

Computer programs: *APEX2* and *SAINT* (Bruker, 2014 ▸), *SHELXT2018/2* (Sheldrick, 2015*a* ▸), *SHELXL2018/3* (Sheldrick, 2015*b* ▸), *ORTEP-3 for Windows* (Farrugia, 2012 ▸) and *CIFTAB* (Sheldrick, 2008 ▸).

## full crystallographic data

### Triacetonitrile(1,4,7-trimethyl-1,4,7-triazacyclononane)cobalt(II) bis(tetraphenylborate) Crystal data

**Table:**

[Co(C_2_H_3_N)_3_(C_9_H_21_N_3_)](C_24_H_20_B)_2_	*F*(000) = 2108
*M**_r_* = 991.80	*D*_x_ = 1.195 Mg m^−^^3^
Monoclinic, *P*2_1_/*c*	Mo *K*α radiation, λ = 0.71073 Å
*a* = 18.1245 (16) Å	Cell parameters from 9821 reflections
*b* = 11.6689 (10) Å	θ = 2.2–24.7°
*c* = 26.067 (2) Å	µ = 0.36 mm^−^^1^
β = 90.332 (2)°	*T* = 133 K
*V* = 5513.0 (8) Å^3^	Platy, orange
*Z* = 4	0.10 × 0.09 × 0.05 mm

### Triacetonitrile(1,4,7-trimethyl-1,4,7-triazacyclononane)cobalt(II) bis(tetraphenylborate) Data collection

**Table:**

Bruker APEXII CCD detector diffractometer	6984 reflections with *I* > 2σ(*I*)
Radiation source: fine-focus sealed tube	*R*_int_ = 0.096
phi and ω scans	θ_max_ = 24.8°, θ_min_ = 1.9°
Absorption correction: multi-scan (SADABS; Krause *et al.*, 2015)	*h* = −21→21
*T*_min_ = 0.627, *T*_max_ = 0.745	*k* = −13→13
112545 measured reflections	*l* = −30→30
9437 independent reflections	

### Triacetonitrile(1,4,7-trimethyl-1,4,7-triazacyclononane)cobalt(II) bis(tetraphenylborate) Refinement

**Table:**

Refinement on *F*^2^	0 restraints
Least-squares matrix: full	Hydrogen site location: inferred from neighbouring sites
*R*[*F*^2^ > 2σ(*F*^2^)] = 0.056	H-atom parameters constrained
*wR*(*F*^2^) = 0.107	*w* = 1/[σ^2^(*F*_o_^2^) + (0.033*P*)^2^ + 3.7116*P*] where *P* = (*F*_o_^2^ + 2*F*_c_^2^)/3
*S* = 1.14	(Δ/σ)_max_ < 0.001
9437 reflections	Δρ_max_ = 0.59 e Å^−^^3^
655 parameters	Δρ_min_ = −0.63 e Å^−^^3^

### Triacetonitrile(1,4,7-trimethyl-1,4,7-triazacyclononane)cobalt(II) bis(tetraphenylborate) Special details

**Table:**

Geometry. All esds (except the esd in the dihedral angle between two l.s. planes) are estimated using the full covariance matrix. The cell esds are taken into account individually in the estimation of esds in distances, angles and torsion angles; correlations between esds in cell parameters are only used when they are defined by crystal symmetry. An approximate (isotropic) treatment of cell esds is used for estimating esds involving l.s. planes.
Refinement. All H atoms were positioned with idealized geometry and refined isotropically with *U*_iso_(H) = 1.2*U*_eq_(C) using a riding model.

### Triacetonitrile(1,4,7-trimethyl-1,4,7-triazacyclononane)cobalt(II) bis(tetraphenylborate) Fractional atomic coordinates and isotropic or equivalent isotropic displacement parameters (Å^2^)

**Table:**

	*x*	*y*	*z*	*U*_iso_*/*U*_eq_	
Co1	0.26079 (2)	0.21794 (3)	0.37198 (2)	0.02127 (11)	
N1	0.36590 (12)	0.2774 (2)	0.34576 (9)	0.0289 (6)	
N2	0.32689 (13)	0.1042 (2)	0.41730 (9)	0.0364 (6)	
N3	0.27676 (13)	0.09052 (19)	0.31402 (8)	0.0297 (6)	
N4	0.25061 (14)	0.3410 (2)	0.43183 (9)	0.0373 (6)	
N5	0.20270 (13)	0.3280 (2)	0.32282 (9)	0.0324 (6)	
N6	0.15961 (13)	0.1475 (2)	0.40119 (9)	0.0302 (6)	
C1	0.37595 (19)	0.4025 (3)	0.34969 (14)	0.0500 (9)	
H1A	0.422038	0.424526	0.332853	0.075*	
H1B	0.378075	0.424796	0.385918	0.075*	
H1C	0.334415	0.441435	0.332902	0.075*	
C10	0.23835 (17)	0.4038 (3)	0.46362 (12)	0.0376 (8)	
C2	0.42496 (16)	0.2172 (3)	0.37525 (12)	0.0445 (8)	
H2A	0.465850	0.271200	0.382424	0.053*	
H2B	0.444754	0.153452	0.354361	0.053*	
C11	0.2220 (2)	0.4837 (3)	0.50480 (13)	0.0664 (12)	
H11A	0.219402	0.561709	0.490959	0.100*	
H11B	0.260938	0.479669	0.530955	0.100*	
H11C	0.174521	0.463643	0.520243	0.100*	
C12	0.17103 (16)	0.3839 (3)	0.29450 (11)	0.0292 (7)	
C13	0.13107 (17)	0.4524 (3)	0.25734 (11)	0.0375 (8)	
H13A	0.089775	0.490987	0.274242	0.056*	
H13B	0.112109	0.402832	0.229941	0.056*	
H13C	0.164259	0.509904	0.242679	0.056*	
C14	0.11375 (16)	0.1172 (3)	0.42739 (11)	0.0292 (7)	
C15	0.05684 (17)	0.0781 (3)	0.46240 (11)	0.0408 (8)	
H15A	0.013637	0.128266	0.459471	0.061*	
H15B	0.075843	0.080410	0.497662	0.061*	
H15C	0.042689	−0.000565	0.453668	0.061*	
C16	0.00729 (16)	0.8301 (3)	0.37025 (10)	0.0298 (7)	
C17	−0.06356 (17)	0.8249 (3)	0.39156 (11)	0.0399 (8)	
H17	−0.079004	0.755634	0.407378	0.048*	
C18	−0.11219 (19)	0.9176 (3)	0.39040 (13)	0.0514 (10)	
H18	−0.159485	0.910873	0.405629	0.062*	
C19	−0.0918 (2)	1.0189 (3)	0.36720 (13)	0.0546 (11)	
H19	−0.124900	1.081956	0.366144	0.066*	
C20	−0.0233 (2)	1.0274 (3)	0.34574 (13)	0.0489 (9)	
H20	−0.008721	1.096751	0.329605	0.059*	
C21	0.02505 (17)	0.9348 (3)	0.34749 (11)	0.0369 (8)	
H21	0.072390	0.943247	0.332497	0.044*	
C22	0.02576 (14)	0.5992 (2)	0.36243 (10)	0.0242 (6)	
C23	0.05553 (15)	0.4955 (2)	0.37991 (10)	0.0280 (7)	
H23	0.096128	0.498067	0.403093	0.034*	
C24	0.02860 (16)	0.3893 (3)	0.36499 (11)	0.0307 (7)	
H24	0.050531	0.321261	0.378052	0.037*	
C25	−0.02994 (16)	0.3825 (3)	0.33126 (11)	0.0343 (7)	
H25	−0.048891	0.310153	0.320958	0.041*	
C26	−0.06053 (16)	0.4821 (3)	0.31272 (12)	0.0357 (7)	
H26	−0.100747	0.478443	0.289238	0.043*	
C27	−0.03319 (15)	0.5880 (2)	0.32799 (11)	0.0304 (7)	
H27	−0.055402	0.655411	0.314519	0.037*	
C28	0.09238 (16)	0.7265 (3)	0.43650 (10)	0.0316 (7)	
C29	0.14978 (18)	0.7970 (3)	0.45322 (11)	0.0398 (8)	
H29	0.175407	0.841343	0.428474	0.048*	
C30	0.1714 (2)	0.8056 (3)	0.50445 (12)	0.0496 (9)	
H30	0.210939	0.854754	0.513967	0.060*	
C31	0.1354 (2)	0.7431 (3)	0.54106 (12)	0.0546 (10)	
H31	0.150092	0.747998	0.576031	0.065*	
C32	0.0779 (2)	0.6732 (3)	0.52669 (12)	0.0555 (10)	
H32	0.052427	0.629733	0.551826	0.067*	
C33	0.05695 (18)	0.6660 (3)	0.47559 (11)	0.0441 (9)	
H33	0.016674	0.617682	0.466679	0.053*	
C34	0.13362 (14)	0.7270 (2)	0.33569 (10)	0.0247 (6)	
C35	0.12318 (15)	0.7591 (2)	0.28431 (10)	0.0295 (7)	
H35	0.075907	0.785862	0.273925	0.035*	
C36	0.17851 (16)	0.7535 (2)	0.24803 (11)	0.0349 (8)	
H36	0.168751	0.776946	0.213751	0.042*	
C37	0.24763 (16)	0.7143 (3)	0.26137 (12)	0.0363 (8)	
H37	0.285910	0.711189	0.236663	0.044*	
C38	0.26038 (16)	0.6794 (2)	0.31121 (12)	0.0352 (8)	
H38	0.307613	0.651257	0.320943	0.042*	
C39	0.20450 (15)	0.6854 (2)	0.34707 (11)	0.0306 (7)	
H39	0.214621	0.660199	0.381041	0.037*	
C40	0.51258 (14)	0.7520 (2)	0.31920 (10)	0.0215 (6)	
C41	0.49323 (14)	0.6505 (2)	0.29379 (10)	0.0256 (6)	
H41	0.507706	0.579698	0.308791	0.031*	
C42	0.45418 (14)	0.6477 (2)	0.24806 (10)	0.0261 (7)	
H42	0.442318	0.576246	0.232589	0.031*	
C43	0.43240 (14)	0.7490 (2)	0.22484 (10)	0.0249 (7)	
H43	0.407080	0.748123	0.192831	0.030*	
C44	0.44819 (14)	0.8509 (2)	0.24909 (10)	0.0254 (6)	
H44	0.432404	0.921059	0.234134	0.031*	
C45	0.48688 (14)	0.8523 (2)	0.29510 (10)	0.0254 (6)	
H45	0.496444	0.923995	0.311034	0.030*	
C46	0.49871 (15)	0.7112 (2)	0.41940 (10)	0.0246 (6)	
C47	0.52292 (17)	0.6700 (2)	0.46687 (10)	0.0315 (7)	
H47	0.574506	0.662922	0.472701	0.038*	
C48	0.47564 (19)	0.6390 (3)	0.50568 (11)	0.0388 (8)	
H48	0.494909	0.610553	0.537172	0.047*	
C49	0.40053 (19)	0.6491 (3)	0.49892 (11)	0.0414 (8)	
H49	0.367657	0.628399	0.525620	0.050*	
C50	0.37390 (18)	0.6897 (3)	0.45288 (12)	0.0411 (8)	
H50	0.322223	0.697581	0.447692	0.049*	
C51	0.42219 (16)	0.7190 (2)	0.41404 (11)	0.0322 (7)	
H51	0.402379	0.745589	0.382387	0.039*	
C52	0.62601 (15)	0.6606 (2)	0.37250 (9)	0.0251 (6)	
C53	0.70063 (16)	0.6897 (3)	0.36737 (11)	0.0357 (8)	
H53	0.713522	0.768607	0.367493	0.043*	
C54	0.75655 (18)	0.6100 (3)	0.36213 (12)	0.0446 (9)	
H54	0.806155	0.635014	0.358631	0.054*	
C55	0.74065 (19)	0.4944 (3)	0.36195 (11)	0.0447 (9)	
H55	0.778641	0.439427	0.357178	0.054*	
C56	0.66883 (18)	0.4603 (3)	0.36880 (10)	0.0374 (8)	
H56	0.656977	0.381066	0.369708	0.045*	
C57	0.61333 (16)	0.5420 (2)	0.37443 (10)	0.0297 (7)	
H57	0.564322	0.516009	0.379859	0.036*	
C58	0.58565 (15)	0.8834 (2)	0.38696 (10)	0.0248 (6)	
C59	0.63458 (16)	0.9399 (3)	0.35433 (11)	0.0326 (7)	
H59	0.650183	0.901700	0.324077	0.039*	
C60	0.66161 (17)	1.0498 (3)	0.36415 (12)	0.0407 (8)	
H60	0.696074	1.083950	0.341477	0.049*	
C61	0.63802 (18)	1.1086 (3)	0.40693 (12)	0.0406 (8)	
H61	0.656582	1.183091	0.414112	0.049*	
C62	0.58764 (17)	1.0590 (3)	0.43899 (11)	0.0357 (8)	
H62	0.569907	1.099913	0.467905	0.043*	
C63	0.56245 (15)	0.9482 (2)	0.42911 (10)	0.0285 (7)	
H63	0.527914	0.915169	0.452055	0.034*	
B1	0.06470 (17)	0.7221 (3)	0.37633 (12)	0.0270 (7)	
B2	0.55686 (17)	0.7525 (3)	0.37481 (12)	0.0241 (7)	
C3	0.39592 (16)	0.1703 (3)	0.42524 (12)	0.0470 (9)	
H3A	0.433697	0.120075	0.441105	0.056*	
H3B	0.386375	0.234537	0.449104	0.056*	
C4	0.29529 (19)	0.0746 (3)	0.46769 (12)	0.0538 (10)	
H4A	0.331093	0.029267	0.487397	0.081*	
H4B	0.250094	0.029752	0.462629	0.081*	
H4C	0.283707	0.144979	0.486477	0.081*	
C5	0.3405 (2)	−0.0026 (3)	0.38691 (13)	0.0509 (9)	
H5A	0.337486	−0.069866	0.409939	0.061*	
H5B	0.390920	0.000031	0.372569	0.061*	
C6	0.2858 (2)	−0.0163 (3)	0.34395 (13)	0.0464 (9)	
H6A	0.302433	−0.078417	0.320865	0.056*	
H6B	0.237452	−0.039030	0.358283	0.056*	
C7	0.21341 (17)	0.0771 (3)	0.27841 (12)	0.0462 (9)	
H7A	0.220658	0.008683	0.257235	0.069*	
H7B	0.209788	0.144769	0.256266	0.069*	
H7C	0.167867	0.069012	0.298162	0.069*	
C8	0.34430 (16)	0.1195 (2)	0.28411 (11)	0.0330 (7)	
H8A	0.335250	0.103584	0.247305	0.040*	
H8B	0.385719	0.070483	0.295726	0.040*	
C9	0.36514 (16)	0.2438 (2)	0.29074 (11)	0.0325 (7)	
H9A	0.414619	0.256903	0.275954	0.039*	
H9B	0.329423	0.292381	0.271835	0.039*	

### Triacetonitrile(1,4,7-trimethyl-1,4,7-triazacyclononane)cobalt(II) bis(tetraphenylborate) Atomic displacement parameters (Å^2^)

**Table:**

	*U*^11^	*U*^22^	*U*^33^	*U*^12^	*U*^13^	*U*^23^
Co1	0.0208 (2)	0.0226 (2)	0.02047 (19)	0.00069 (17)	0.00212 (14)	0.00164 (17)
N1	0.0234 (13)	0.0275 (13)	0.0360 (14)	−0.0043 (11)	0.0051 (10)	−0.0021 (12)
N2	0.0324 (15)	0.0413 (16)	0.0355 (15)	0.0051 (13)	−0.0033 (12)	0.0076 (12)
N3	0.0344 (14)	0.0260 (14)	0.0288 (13)	−0.0031 (11)	0.0036 (11)	−0.0040 (11)
N4	0.0403 (16)	0.0392 (16)	0.0326 (15)	−0.0025 (13)	0.0073 (12)	−0.0078 (13)
N5	0.0313 (15)	0.0370 (15)	0.0291 (14)	0.0077 (12)	0.0089 (12)	0.0084 (12)
N6	0.0264 (14)	0.0390 (15)	0.0253 (13)	−0.0038 (12)	0.0029 (11)	0.0062 (11)
C1	0.048 (2)	0.035 (2)	0.067 (2)	−0.0204 (17)	0.0163 (18)	−0.0089 (18)
C10	0.041 (2)	0.040 (2)	0.0316 (18)	−0.0055 (16)	0.0042 (15)	−0.0051 (16)
C2	0.0219 (17)	0.057 (2)	0.054 (2)	−0.0013 (17)	−0.0046 (15)	−0.0076 (18)
C11	0.090 (3)	0.062 (3)	0.047 (2)	−0.006 (2)	0.015 (2)	−0.029 (2)
C12	0.0309 (17)	0.0326 (18)	0.0243 (16)	0.0035 (14)	0.0084 (13)	0.0040 (14)
C13	0.046 (2)	0.0399 (19)	0.0263 (16)	0.0097 (16)	0.0008 (14)	0.0089 (14)
C14	0.0300 (17)	0.0325 (18)	0.0250 (16)	−0.0011 (14)	−0.0016 (14)	0.0003 (13)
C15	0.0392 (19)	0.052 (2)	0.0308 (17)	−0.0112 (16)	0.0117 (14)	0.0035 (15)
C16	0.0325 (18)	0.0333 (18)	0.0234 (15)	0.0018 (14)	−0.0018 (13)	−0.0101 (13)
C17	0.040 (2)	0.044 (2)	0.0358 (18)	0.0070 (16)	0.0046 (15)	−0.0113 (15)
C18	0.041 (2)	0.072 (3)	0.041 (2)	0.022 (2)	−0.0032 (16)	−0.027 (2)
C19	0.067 (3)	0.051 (3)	0.046 (2)	0.032 (2)	−0.021 (2)	−0.0253 (19)
C20	0.064 (3)	0.032 (2)	0.051 (2)	0.0128 (18)	−0.0193 (19)	−0.0105 (16)
C21	0.0397 (19)	0.0313 (18)	0.0395 (18)	0.0042 (15)	−0.0068 (15)	−0.0086 (15)
C22	0.0194 (15)	0.0308 (17)	0.0224 (15)	0.0029 (13)	0.0078 (12)	−0.0011 (12)
C23	0.0239 (16)	0.0351 (18)	0.0252 (16)	0.0017 (14)	0.0057 (12)	0.0031 (13)
C24	0.0321 (18)	0.0283 (17)	0.0319 (17)	0.0028 (14)	0.0092 (14)	0.0058 (13)
C25	0.0341 (18)	0.0279 (18)	0.0412 (19)	−0.0011 (15)	0.0068 (15)	−0.0024 (14)
C26	0.0284 (17)	0.0366 (19)	0.0419 (19)	0.0006 (15)	−0.0067 (14)	−0.0037 (15)
C27	0.0301 (17)	0.0269 (17)	0.0343 (17)	0.0037 (14)	0.0023 (14)	0.0014 (13)
C28	0.0349 (17)	0.0292 (17)	0.0308 (16)	0.0085 (15)	0.0023 (13)	−0.0058 (14)
C29	0.058 (2)	0.0297 (18)	0.0313 (17)	0.0019 (16)	−0.0079 (15)	0.0000 (14)
C30	0.075 (3)	0.034 (2)	0.040 (2)	0.0057 (18)	−0.0187 (18)	−0.0065 (16)
C31	0.095 (3)	0.046 (2)	0.0229 (18)	0.019 (2)	−0.0065 (19)	−0.0120 (16)
C32	0.076 (3)	0.063 (3)	0.0279 (19)	0.008 (2)	0.0200 (18)	−0.0055 (18)
C33	0.048 (2)	0.055 (2)	0.0297 (18)	0.0012 (17)	0.0155 (15)	−0.0088 (16)
C34	0.0256 (16)	0.0184 (15)	0.0300 (16)	−0.0022 (13)	−0.0003 (12)	−0.0018 (13)
C35	0.0261 (16)	0.0299 (17)	0.0323 (17)	0.0019 (13)	0.0021 (13)	−0.0006 (13)
C36	0.0357 (18)	0.0365 (19)	0.0325 (17)	−0.0021 (15)	0.0076 (14)	0.0009 (14)
C37	0.0286 (17)	0.0332 (18)	0.047 (2)	−0.0058 (15)	0.0144 (14)	−0.0107 (16)
C38	0.0247 (17)	0.0278 (17)	0.053 (2)	0.0010 (13)	0.0007 (15)	−0.0091 (15)
C39	0.0317 (18)	0.0242 (16)	0.0357 (17)	−0.0007 (13)	−0.0017 (14)	−0.0003 (13)
C40	0.0209 (14)	0.0212 (15)	0.0225 (14)	−0.0007 (12)	0.0078 (11)	0.0009 (11)
C41	0.0280 (16)	0.0218 (16)	0.0271 (16)	−0.0002 (13)	0.0044 (13)	0.0027 (12)
C42	0.0258 (16)	0.0269 (17)	0.0258 (16)	−0.0032 (13)	0.0039 (12)	−0.0047 (13)
C43	0.0217 (15)	0.0335 (18)	0.0195 (14)	0.0004 (13)	0.0029 (11)	−0.0011 (12)
C44	0.0262 (16)	0.0237 (16)	0.0264 (16)	0.0053 (13)	0.0036 (12)	0.0023 (13)
C45	0.0270 (16)	0.0233 (16)	0.0260 (16)	0.0018 (13)	0.0043 (13)	−0.0020 (12)
C46	0.0354 (17)	0.0149 (14)	0.0237 (15)	0.0004 (13)	0.0055 (12)	−0.0033 (12)
C47	0.0427 (19)	0.0285 (17)	0.0233 (16)	−0.0008 (14)	0.0029 (14)	−0.0019 (13)
C48	0.064 (2)	0.0301 (18)	0.0222 (16)	−0.0004 (17)	0.0052 (15)	0.0007 (13)
C49	0.061 (2)	0.0352 (19)	0.0285 (18)	−0.0084 (17)	0.0210 (16)	−0.0005 (14)
C50	0.0385 (19)	0.046 (2)	0.0393 (19)	−0.0042 (16)	0.0145 (15)	0.0001 (16)
C51	0.0381 (18)	0.0297 (17)	0.0290 (16)	0.0009 (15)	0.0058 (13)	0.0027 (14)
C52	0.0346 (17)	0.0275 (16)	0.0132 (13)	0.0027 (13)	0.0016 (12)	0.0004 (12)
C53	0.0365 (19)	0.0368 (19)	0.0337 (17)	0.0022 (15)	0.0021 (14)	0.0013 (14)
C54	0.0337 (19)	0.058 (2)	0.042 (2)	0.0105 (18)	0.0036 (15)	0.0015 (17)
C55	0.047 (2)	0.056 (2)	0.0312 (18)	0.0261 (19)	−0.0004 (16)	−0.0026 (16)
C56	0.058 (2)	0.0317 (18)	0.0225 (16)	0.0131 (17)	−0.0064 (15)	0.0002 (13)
C57	0.0377 (18)	0.0314 (18)	0.0199 (15)	0.0023 (15)	−0.0011 (13)	0.0019 (13)
C58	0.0280 (16)	0.0249 (16)	0.0215 (15)	0.0012 (13)	−0.0052 (12)	0.0041 (12)
C59	0.0366 (18)	0.0323 (18)	0.0287 (16)	−0.0026 (15)	−0.0041 (14)	0.0061 (14)
C60	0.041 (2)	0.037 (2)	0.044 (2)	−0.0097 (16)	−0.0089 (16)	0.0191 (16)
C61	0.052 (2)	0.0230 (17)	0.047 (2)	−0.0048 (16)	−0.0231 (17)	0.0055 (15)
C62	0.047 (2)	0.0253 (17)	0.0344 (18)	0.0056 (15)	−0.0154 (15)	−0.0024 (14)
C63	0.0336 (17)	0.0247 (16)	0.0273 (16)	0.0010 (14)	−0.0069 (13)	0.0038 (13)
B1	0.0265 (18)	0.0271 (18)	0.0274 (17)	0.0029 (16)	0.0015 (14)	−0.0014 (15)
B2	0.0295 (18)	0.0183 (17)	0.0244 (17)	−0.0005 (14)	0.0027 (14)	0.0007 (13)
C3	0.0270 (18)	0.072 (3)	0.042 (2)	0.0050 (17)	−0.0111 (15)	0.0013 (18)
C4	0.050 (2)	0.073 (3)	0.038 (2)	0.010 (2)	−0.0079 (16)	0.0277 (19)
C5	0.058 (2)	0.033 (2)	0.061 (2)	0.0158 (17)	0.0042 (19)	0.0135 (17)
C6	0.066 (2)	0.0246 (18)	0.048 (2)	−0.0005 (17)	0.0122 (18)	−0.0017 (15)
C7	0.046 (2)	0.059 (2)	0.0336 (18)	−0.0164 (18)	−0.0016 (16)	−0.0149 (17)
C8	0.0333 (17)	0.0351 (18)	0.0305 (17)	0.0034 (14)	0.0088 (13)	−0.0077 (14)
C9	0.0264 (16)	0.0389 (19)	0.0325 (17)	−0.0005 (14)	0.0150 (13)	0.0017 (14)

### Triacetonitrile(1,4,7-trimethyl-1,4,7-triazacyclononane)cobalt(II) bis(tetraphenylborate) Geometric parameters (Å, º)

**Table:**

Co1—N1	2.143 (2)	C35—C36	1.384 (4)
Co1—N2	2.139 (2)	C35—H35	0.9500
Co1—N3	2.141 (2)	C36—C37	1.377 (4)
Co1—N4	2.129 (3)	C36—H36	0.9500
Co1—N5	2.094 (3)	C37—C38	1.380 (4)
Co1—N6	2.153 (2)	C37—H37	0.9500
N1—C1	1.475 (4)	C38—C39	1.384 (4)
N1—C9	1.487 (3)	C38—H38	0.9500
N1—C2	1.490 (4)	C39—H39	0.9500
N2—C4	1.477 (4)	C40—C41	1.401 (4)
N2—C3	1.483 (4)	C40—C45	1.406 (4)
N2—C5	1.498 (4)	C40—B2	1.653 (4)
N3—C6	1.479 (4)	C41—C42	1.383 (4)
N3—C7	1.480 (4)	C41—H41	0.9500
N3—C8	1.494 (3)	C42—C43	1.384 (4)
N4—C10	1.129 (4)	C42—H42	0.9500
N5—C12	1.138 (3)	C43—C44	1.376 (4)
N6—C14	1.135 (3)	C43—H43	0.9500
C1—H1A	0.9800	C44—C45	1.386 (4)
C1—H1B	0.9800	C44—H44	0.9500
C1—H1C	0.9800	C45—H45	0.9500
C10—C11	1.454 (4)	C46—C51	1.396 (4)
C2—C3	1.511 (4)	C46—C47	1.396 (4)
C2—H2A	0.9900	C46—B2	1.645 (4)
C2—H2B	0.9900	C47—C48	1.378 (4)
C11—H11A	0.9800	C47—H47	0.9500
C11—H11B	0.9800	C48—C49	1.377 (4)
C11—H11C	0.9800	C48—H48	0.9500
C12—C13	1.447 (4)	C49—C50	1.375 (4)
C13—H13A	0.9800	C49—H49	0.9500
C13—H13B	0.9800	C50—C51	1.385 (4)
C13—H13C	0.9800	C50—H50	0.9500
C14—C15	1.455 (4)	C51—H51	0.9500
C15—H15A	0.9800	C52—C53	1.402 (4)
C15—H15B	0.9800	C52—C57	1.404 (4)
C15—H15C	0.9800	C52—B2	1.651 (4)
C16—C21	1.397 (4)	C53—C54	1.383 (4)
C16—C17	1.403 (4)	C53—H53	0.9500
C16—B1	1.642 (4)	C54—C55	1.379 (5)
C17—C18	1.395 (4)	C54—H54	0.9500
C17—H17	0.9500	C55—C56	1.374 (4)
C18—C19	1.379 (5)	C55—H55	0.9500
C18—H18	0.9500	C56—C57	1.394 (4)
C19—C20	1.368 (5)	C56—H56	0.9500
C19—H19	0.9500	C57—H57	0.9500
C20—C21	1.392 (4)	C58—C59	1.398 (4)
C20—H20	0.9500	C58—C63	1.401 (4)
C21—H21	0.9500	C58—B2	1.644 (4)
C22—C27	1.398 (4)	C59—C60	1.396 (4)
C22—C23	1.399 (4)	C59—H59	0.9500
C22—B1	1.638 (4)	C60—C61	1.379 (4)
C23—C24	1.387 (4)	C60—H60	0.9500
C23—H23	0.9500	C61—C62	1.370 (4)
C24—C25	1.376 (4)	C61—H61	0.9500
C24—H24	0.9500	C62—C63	1.394 (4)
C25—C26	1.374 (4)	C62—H62	0.9500
C25—H25	0.9500	C63—H63	0.9500
C26—C27	1.389 (4)	C3—H3A	0.9900
C26—H26	0.9500	C3—H3B	0.9900
C27—H27	0.9500	C4—H4A	0.9800
C28—C29	1.394 (4)	C4—H4B	0.9800
C28—C33	1.399 (4)	C4—H4C	0.9800
C28—B1	1.645 (4)	C5—C6	1.500 (5)
C29—C30	1.393 (4)	C5—H5A	0.9900
C29—H29	0.9500	C5—H5B	0.9900
C30—C31	1.369 (5)	C6—H6A	0.9900
C30—H30	0.9500	C6—H6B	0.9900
C31—C32	1.374 (5)	C7—H7A	0.9800
C31—H31	0.9500	C7—H7B	0.9800
C32—C33	1.385 (4)	C7—H7C	0.9800
C32—H32	0.9500	C8—C9	1.508 (4)
C33—H33	0.9500	C8—H8A	0.9900
C34—C35	1.403 (4)	C8—H8B	0.9900
C34—C39	1.403 (4)	C9—H9A	0.9900
C34—B1	1.643 (4)	C9—H9B	0.9900
			
N5—Co1—N4	89.38 (10)	C38—C37—H37	120.6
N4—Co1—N2	93.69 (10)	C37—C38—C39	120.1 (3)
N5—Co1—N3	93.66 (9)	C37—C38—H38	119.9
N5—Co1—N1	92.93 (9)	C39—C38—H38	119.9
N4—Co1—N1	95.53 (9)	C38—C39—C34	123.2 (3)
N1—Co1—N2	83.16 (9)	C38—C39—H39	118.4
N1—Co1—N3	82.86 (9)	C34—C39—H39	118.4
N2—Co1—N3	83.18 (9)	C41—C40—C45	114.3 (2)
N1—Co1—N6	175.58 (9)	C41—C40—B2	122.4 (2)
N2—Co1—N5	175.23 (9)	C45—C40—B2	123.2 (2)
N3—Co1—N4	176.62 (10)	C42—C41—C40	123.6 (3)
N5—Co1—N6	91.39 (9)	C42—C41—H41	118.2
N4—Co1—N6	85.44 (9)	C40—C41—H41	118.2
N2—Co1—N6	92.49 (9)	C41—C42—C43	120.0 (3)
N3—Co1—N6	95.94 (9)	C41—C42—H42	120.0
C1—N1—C9	109.2 (2)	C43—C42—H42	120.0
C1—N1—C2	110.1 (2)	C44—C43—C42	118.6 (3)
C9—N1—C2	112.1 (2)	C44—C43—H43	120.7
C1—N1—Co1	114.07 (18)	C42—C43—H43	120.7
C9—N1—Co1	102.65 (16)	C43—C44—C45	120.7 (3)
C2—N1—Co1	108.68 (17)	C43—C44—H44	119.7
C4—N2—C3	109.2 (2)	C45—C44—H44	119.7
C4—N2—C5	110.0 (3)	C44—C45—C40	122.8 (3)
C3—N2—C5	111.4 (2)	C44—C45—H45	118.6
C4—N2—Co1	114.68 (19)	C40—C45—H45	118.6
C3—N2—Co1	102.92 (18)	C51—C46—C47	114.7 (2)
C5—N2—Co1	108.51 (18)	C51—C46—B2	123.4 (2)
C6—N3—C7	108.9 (2)	C47—C46—B2	121.8 (2)
C6—N3—C8	112.2 (2)	C48—C47—C46	123.2 (3)
C7—N3—C8	109.4 (2)	C48—C47—H47	118.4
C6—N3—Co1	103.20 (17)	C46—C47—H47	118.4
C7—N3—Co1	114.11 (18)	C49—C48—C47	120.1 (3)
C8—N3—Co1	109.00 (16)	C49—C48—H48	119.9
C10—N4—Co1	173.5 (3)	C47—C48—H48	119.9
C12—N5—Co1	176.8 (2)	C50—C49—C48	118.9 (3)
C14—N6—Co1	163.7 (2)	C50—C49—H49	120.6
N1—C1—H1A	109.5	C48—C49—H49	120.6
N1—C1—H1B	109.5	C49—C50—C51	120.2 (3)
H1A—C1—H1B	109.5	C49—C50—H50	119.9
N1—C1—H1C	109.5	C51—C50—H50	119.9
H1A—C1—H1C	109.5	C50—C51—C46	122.8 (3)
H1B—C1—H1C	109.5	C50—C51—H51	118.6
N4—C10—C11	179.4 (4)	C46—C51—H51	118.6
N1—C2—C3	111.3 (2)	C53—C52—C57	113.6 (3)
N1—C2—H2A	109.4	C53—C52—B2	125.4 (3)
C3—C2—H2A	109.4	C57—C52—B2	121.0 (2)
N1—C2—H2B	109.4	C54—C53—C52	123.7 (3)
C3—C2—H2B	109.4	C54—C53—H53	118.1
H2A—C2—H2B	108.0	C52—C53—H53	118.1
C10—C11—H11A	109.5	C55—C54—C53	120.3 (3)
C10—C11—H11B	109.5	C55—C54—H54	119.8
H11A—C11—H11B	109.5	C53—C54—H54	119.8
C10—C11—H11C	109.5	C56—C55—C54	118.7 (3)
H11A—C11—H11C	109.5	C56—C55—H55	120.6
H11B—C11—H11C	109.5	C54—C55—H55	120.6
N5—C12—C13	178.3 (3)	C55—C56—C57	120.0 (3)
C12—C13—H13A	109.5	C55—C56—H56	120.0
C12—C13—H13B	109.5	C57—C56—H56	120.0
H13A—C13—H13B	109.5	C56—C57—C52	123.5 (3)
C12—C13—H13C	109.5	C56—C57—H57	118.3
H13A—C13—H13C	109.5	C52—C57—H57	118.3
H13B—C13—H13C	109.5	C59—C58—C63	114.7 (3)
N6—C14—C15	178.0 (3)	C59—C58—B2	121.5 (2)
C14—C15—H15A	109.5	C63—C58—B2	123.8 (2)
C14—C15—H15B	109.5	C60—C59—C58	123.0 (3)
H15A—C15—H15B	109.5	C60—C59—H59	118.5
C14—C15—H15C	109.5	C58—C59—H59	118.5
H15A—C15—H15C	109.5	C61—C60—C59	119.7 (3)
H15B—C15—H15C	109.5	C61—C60—H60	120.2
C21—C16—C17	114.8 (3)	C59—C60—H60	120.2
C21—C16—B1	124.4 (3)	C62—C61—C60	119.6 (3)
C17—C16—B1	120.6 (3)	C62—C61—H61	120.2
C18—C17—C16	122.5 (3)	C60—C61—H61	120.2
C18—C17—H17	118.8	C61—C62—C63	119.9 (3)
C16—C17—H17	118.8	C61—C62—H62	120.1
C19—C18—C17	120.2 (3)	C63—C62—H62	120.1
C19—C18—H18	119.9	C62—C63—C58	123.1 (3)
C17—C18—H18	119.9	C62—C63—H63	118.5
C20—C19—C18	119.2 (3)	C58—C63—H63	118.5
C20—C19—H19	120.4	C22—B1—C16	112.3 (2)
C18—C19—H19	120.4	C22—B1—C34	102.5 (2)
C19—C20—C21	120.2 (3)	C16—B1—C34	113.3 (2)
C19—C20—H20	119.9	C22—B1—C28	111.5 (2)
C21—C20—H20	119.9	C16—B1—C28	104.9 (2)
C20—C21—C16	123.1 (3)	C34—B1—C28	112.6 (2)
C20—C21—H21	118.4	C58—B2—C46	109.9 (2)
C16—C21—H21	118.4	C58—B2—C52	111.7 (2)
C27—C22—C23	114.9 (3)	C46—B2—C52	109.0 (2)
C27—C22—B1	123.4 (3)	C58—B2—C40	108.9 (2)
C23—C22—B1	121.3 (2)	C46—B2—C40	108.0 (2)
C24—C23—C22	123.1 (3)	C52—B2—C40	109.3 (2)
C24—C23—H23	118.4	N2—C3—C2	111.5 (3)
C22—C23—H23	118.4	N2—C3—H3A	109.3
C25—C24—C23	119.9 (3)	C2—C3—H3A	109.3
C25—C24—H24	120.0	N2—C3—H3B	109.3
C23—C24—H24	120.0	C2—C3—H3B	109.3
C26—C25—C24	119.0 (3)	H3A—C3—H3B	108.0
C26—C25—H25	120.5	N2—C4—H4A	109.5
C24—C25—H25	120.5	N2—C4—H4B	109.5
C25—C26—C27	120.6 (3)	H4A—C4—H4B	109.5
C25—C26—H26	119.7	N2—C4—H4C	109.5
C27—C26—H26	119.7	H4A—C4—H4C	109.5
C26—C27—C22	122.5 (3)	H4B—C4—H4C	109.5
C26—C27—H27	118.7	N2—C5—C6	112.0 (3)
C22—C27—H27	118.7	N2—C5—H5A	109.2
C29—C28—C33	114.5 (3)	C6—C5—H5A	109.2
C29—C28—B1	122.6 (3)	N2—C5—H5B	109.2
C33—C28—B1	122.7 (3)	C6—C5—H5B	109.2
C30—C29—C28	123.2 (3)	H5A—C5—H5B	107.9
C30—C29—H29	118.4	N3—C6—C5	111.9 (3)
C28—C29—H29	118.4	N3—C6—H6A	109.2
C31—C30—C29	119.8 (3)	C5—C6—H6A	109.2
C31—C30—H30	120.1	N3—C6—H6B	109.2
C29—C30—H30	120.1	C5—C6—H6B	109.2
C30—C31—C32	119.3 (3)	H6A—C6—H6B	107.9
C30—C31—H31	120.3	N3—C7—H7A	109.5
C32—C31—H31	120.3	N3—C7—H7B	109.5
C31—C32—C33	120.1 (3)	H7A—C7—H7B	109.5
C31—C32—H32	120.0	N3—C7—H7C	109.5
C33—C32—H32	120.0	H7A—C7—H7C	109.5
C32—C33—C28	123.1 (3)	H7B—C7—H7C	109.5
C32—C33—H33	118.5	N3—C8—C9	111.3 (2)
C28—C33—H33	118.5	N3—C8—H8A	109.4
C35—C34—C39	114.4 (2)	C9—C8—H8A	109.4
C35—C34—B1	121.7 (2)	N3—C8—H8B	109.4
C39—C34—B1	123.4 (2)	C9—C8—H8B	109.4
C36—C35—C34	123.1 (3)	H8A—C8—H8B	108.0
C36—C35—H35	118.5	N1—C9—C8	111.4 (2)
C34—C35—H35	118.5	N1—C9—H9A	109.3
C37—C36—C35	120.3 (3)	C8—C9—H9A	109.3
C37—C36—H36	119.8	N1—C9—H9B	109.3
C35—C36—H36	119.8	C8—C9—H9B	109.3
C36—C37—C38	118.9 (3)	H9A—C9—H9B	108.0
C36—C37—H37	120.6		
			
C1—N1—C2—C3	104.4 (3)	C27—C22—B1—C16	28.2 (3)
C9—N1—C2—C3	−133.9 (3)	C23—C22—B1—C16	−159.6 (2)
Co1—N1—C2—C3	−21.2 (3)	C27—C22—B1—C34	−93.6 (3)
C21—C16—C17—C18	−0.5 (4)	C23—C22—B1—C34	78.6 (3)
B1—C16—C17—C18	175.4 (3)	C27—C22—B1—C28	145.7 (2)
C16—C17—C18—C19	0.8 (5)	C23—C22—B1—C28	−42.2 (3)
C17—C18—C19—C20	−0.4 (5)	C21—C16—B1—C22	−137.0 (3)
C18—C19—C20—C21	−0.1 (5)	C17—C16—B1—C22	47.6 (3)
C19—C20—C21—C16	0.4 (5)	C21—C16—B1—C34	−21.5 (4)
C17—C16—C21—C20	−0.1 (4)	C17—C16—B1—C34	163.0 (3)
B1—C16—C21—C20	−175.8 (3)	C21—C16—B1—C28	101.7 (3)
C27—C22—C23—C24	−0.7 (4)	C17—C16—B1—C28	−73.7 (3)
B1—C22—C23—C24	−173.5 (2)	C35—C34—B1—C22	79.8 (3)
C22—C23—C24—C25	0.3 (4)	C39—C34—B1—C22	−91.3 (3)
C23—C24—C25—C26	0.3 (4)	C35—C34—B1—C16	−41.3 (4)
C24—C25—C26—C27	−0.4 (4)	C39—C34—B1—C16	147.6 (3)
C25—C26—C27—C22	−0.1 (4)	C35—C34—B1—C28	−160.2 (3)
C23—C22—C27—C26	0.6 (4)	C39—C34—B1—C28	28.7 (4)
B1—C22—C27—C26	173.2 (3)	C29—C28—B1—C22	154.6 (3)
C33—C28—C29—C30	1.1 (5)	C33—C28—B1—C22	−30.0 (4)
B1—C28—C29—C30	176.8 (3)	C29—C28—B1—C16	−83.6 (3)
C28—C29—C30—C31	−0.2 (5)	C33—C28—B1—C16	91.8 (3)
C29—C30—C31—C32	−0.5 (5)	C29—C28—B1—C34	40.0 (4)
C30—C31—C32—C33	0.3 (5)	C33—C28—B1—C34	−144.6 (3)
C31—C32—C33—C28	0.7 (5)	C59—C58—B2—C46	−178.8 (2)
C29—C28—C33—C32	−1.3 (5)	C63—C58—B2—C46	−0.6 (4)
B1—C28—C33—C32	−177.1 (3)	C59—C58—B2—C52	60.2 (3)
C39—C34—C35—C36	−1.8 (4)	C63—C58—B2—C52	−121.6 (3)
B1—C34—C35—C36	−173.6 (3)	C59—C58—B2—C40	−60.7 (3)
C34—C35—C36—C37	0.6 (4)	C63—C58—B2—C40	117.5 (3)
C35—C36—C37—C38	0.7 (4)	C51—C46—B2—C58	96.3 (3)
C36—C37—C38—C39	−0.7 (4)	C47—C46—B2—C58	−80.8 (3)
C37—C38—C39—C34	−0.6 (4)	C51—C46—B2—C52	−141.0 (3)
C35—C34—C39—C38	1.8 (4)	C47—C46—B2—C52	41.9 (3)
B1—C34—C39—C38	173.5 (3)	C51—C46—B2—C40	−22.4 (3)
C45—C40—C41—C42	1.9 (4)	C47—C46—B2—C40	160.5 (2)
B2—C40—C41—C42	177.9 (2)	C53—C52—B2—C58	−16.4 (4)
C40—C41—C42—C43	0.3 (4)	C57—C52—B2—C58	165.2 (2)
C41—C42—C43—C44	−2.2 (4)	C53—C52—B2—C46	−137.9 (3)
C42—C43—C44—C45	1.8 (4)	C57—C52—B2—C46	43.6 (3)
C43—C44—C45—C40	0.6 (4)	C53—C52—B2—C40	104.2 (3)
C41—C40—C45—C44	−2.4 (4)	C57—C52—B2—C40	−74.2 (3)
B2—C40—C45—C44	−178.4 (2)	C41—C40—B2—C58	168.1 (2)
C51—C46—C47—C48	0.1 (4)	C45—C40—B2—C58	−16.3 (3)
B2—C46—C47—C48	177.4 (3)	C41—C40—B2—C46	−72.7 (3)
C46—C47—C48—C49	−0.7 (5)	C45—C40—B2—C46	102.9 (3)
C47—C48—C49—C50	0.5 (5)	C41—C40—B2—C52	45.7 (3)
C48—C49—C50—C51	0.4 (5)	C45—C40—B2—C52	−138.6 (2)
C49—C50—C51—C46	−1.1 (5)	C4—N2—C3—C2	−172.0 (3)
C47—C46—C51—C50	0.8 (4)	C5—N2—C3—C2	66.3 (3)
B2—C46—C51—C50	−176.5 (3)	Co1—N2—C3—C2	−49.8 (3)
C57—C52—C53—C54	3.0 (4)	N1—C2—C3—N2	49.6 (4)
B2—C52—C53—C54	−175.5 (3)	C4—N2—C5—C6	106.9 (3)
C52—C53—C54—C55	−0.3 (5)	C3—N2—C5—C6	−131.9 (3)
C53—C54—C55—C56	−2.2 (5)	Co1—N2—C5—C6	−19.3 (3)
C54—C55—C56—C57	1.7 (4)	C7—N3—C6—C5	−170.4 (3)
C55—C56—C57—C52	1.3 (4)	C8—N3—C6—C5	68.4 (3)
C53—C52—C57—C56	−3.5 (4)	Co1—N3—C6—C5	−48.8 (3)
B2—C52—C57—C56	175.0 (2)	N2—C5—C6—N3	47.6 (4)
C63—C58—C59—C60	3.3 (4)	C6—N3—C8—C9	−132.8 (3)
B2—C58—C59—C60	−178.3 (3)	C7—N3—C8—C9	106.2 (3)
C58—C59—C60—C61	−2.1 (5)	Co1—N3—C8—C9	−19.1 (3)
C59—C60—C61—C62	−0.8 (5)	C1—N1—C9—C8	−171.9 (2)
C60—C61—C62—C63	2.1 (4)	C2—N1—C9—C8	65.9 (3)
C61—C62—C63—C58	−0.7 (4)	Co1—N1—C9—C8	−50.6 (2)
C59—C58—C63—C62	−2.0 (4)	N3—C8—C9—N1	48.6 (3)
B2—C58—C63—C62	179.7 (3)		
