# Supplementary material for: Triaceto­nitrile­(1,4,7-trimethyl-1,4,7-tri­aza­cyclonona­ne)cobalt(II) bis­(tetra­phenyl­borate)
Source: IUCrdata. 2024 Jun 11;9(Pt 6):x240539. doi: 10.1107/S241431462400539X (PMC11223681; doi:10.1107/S241431462400539X)
Supplement: Supplementary file 3 [file x-09-x240539-sup3.pdf]

# Supporting Information for Synthesis, crystal structure of $[(tacn)Co(NCMe)_3][BPh_4]_2$

Jonghoon Choi *
[1]Department of Chemistry Education, Chonnam National University, Gwangju 61186, Republic of Korea

**Contents**

**Experimental Section**
**Figure S1.** $^{1}H$ NMR spectrum of $[(tacn)Co(NCMe)_3][BPh_4]_2$ **1** in DMSO-$d_6$.
**Figure S2.** $^{11}B$ NMR spectrum of $[(tacn)Co(NCMe)_3][BPh_4]_2$ **1** in DMSO-$d_6$.
**Figure S3.** Solid-state structure of **1**.
**Table S4.** Selected bond distances and angles for **1**.

**Figure S1.** $^{1}H$ NMR spectrum of $[(tacn)Co(NCMe)_3][BPh_4]_2$ (**1**) in DMSO-$d_6$ at room temperature.

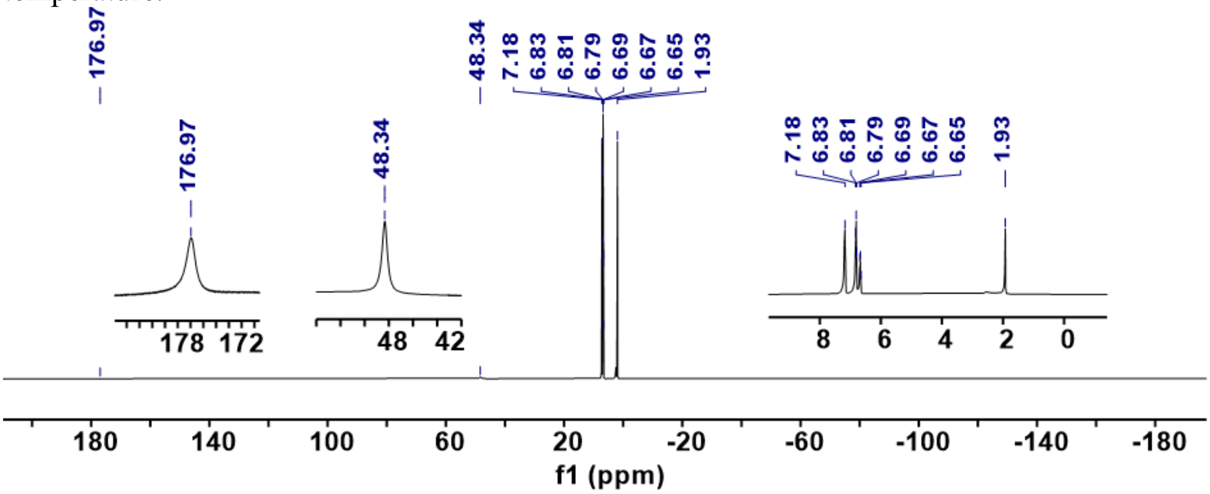


**Figure S2.** $^{11}B$ NMR spectrum of $[(tacn)Co(NCMe)_3][BPh_4]_2$ (**1**) in DMSO-$d_6$ at room temperature.

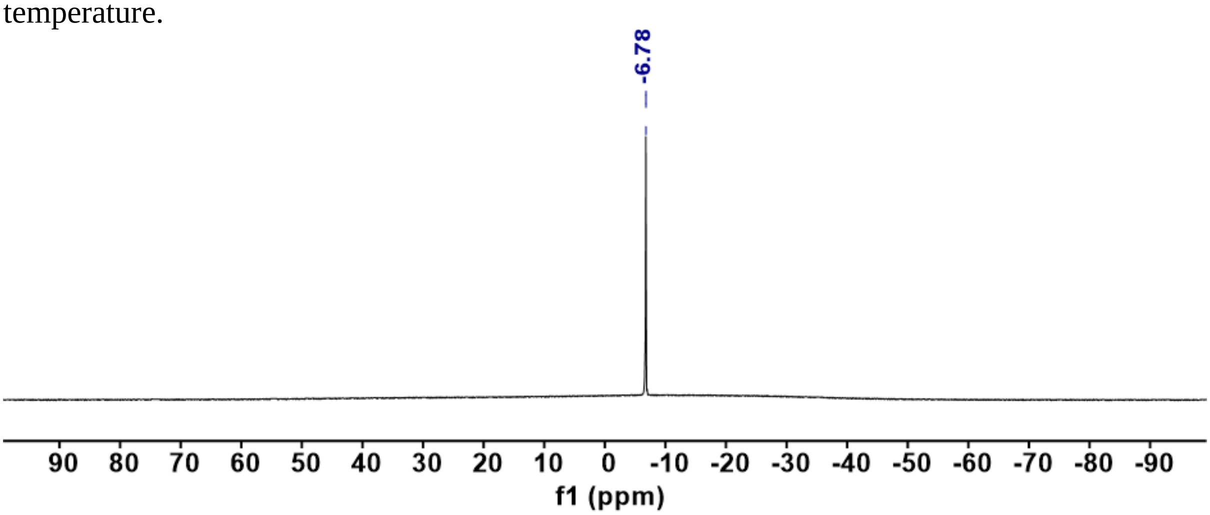

**Figure S3.** Solid-state structure of **1**.

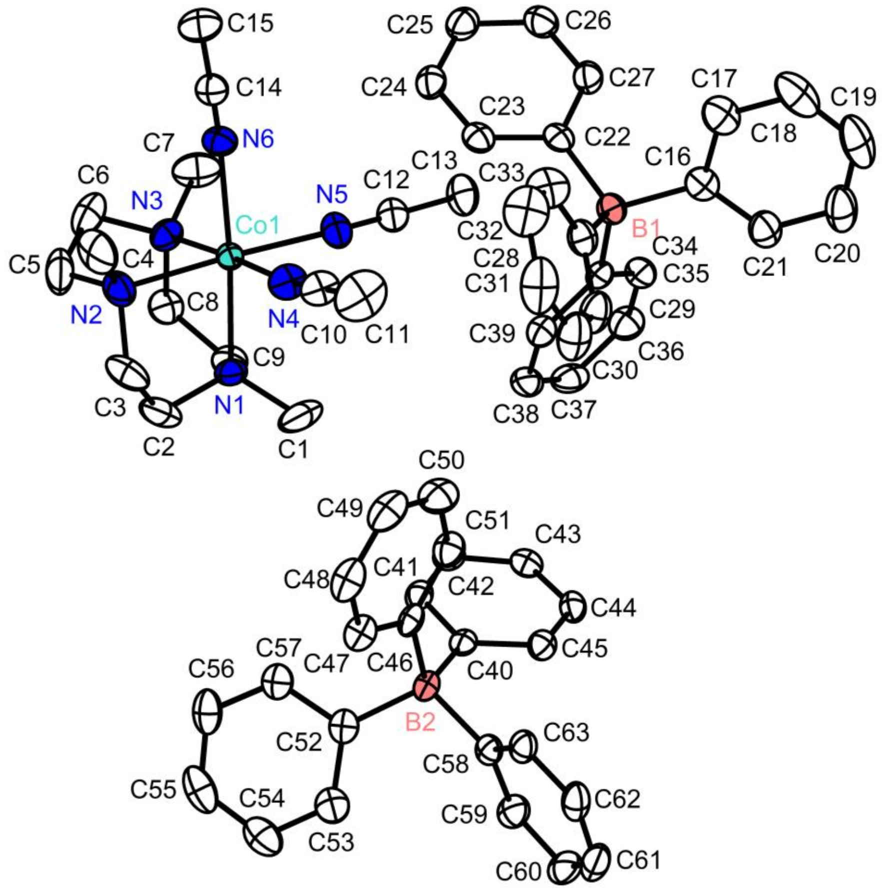


| | | Bond angle | |
|---|---|---|---|
| $d_{Co1\text{-}P1}$ | 2.2258(9) | ∠P1-Co1-P2 | 167.00(3) |
| $d_{Co1\text{-}P2}$ | 2.2272(8) | ∠N1-Co1-O1 | 177.0(1) |
| $d_{Co1\text{-}N1}$ | 1.899(2) | ∠Co1-O1-C27 | 114.2(3) |
| $d_{Co1\text{-}O1}$ | 1.928(2) | ∠O1-C27-O2 | 126.8(4) |
| $d_{C27\text{-}O1}$ | 1.237(5) | | |
| $d_{C27\text{-}O2}$ | 1.233(6) | | |
